# A Collaborative-Care Telephone-Based Intervention for Depression, Anxiety, and at-Risk Drinking in Primary Care: The PARTNERs Randomized Clinical Trial

**DOI:** 10.1177/07067437231156243

**Published:** 2023-02-28

**Authors:** M. Ishrat Husain, David J. Rodie, Athina Perivolaris, Marcos Sanches, Allison Crawford, Kyle P. Fitzgibbon, Andrea Levinson, Rose Geist, Paul Kurdyak, Brian Mitchell, David Oslin, Nadiya Sunderji, Benoit H. Mulsant, for the PARTNERs Study Group

**Affiliations:** 1 7978 Centre for Addiction and Mental Health (CAMH), Toronto, ON, Canada; 2 Department of Psychiatry, Temerty Faculty of Medicine, 7938 University of Toronto, Toronto, ON, Canada; 3 7979 Hospital for Sick Children, Toronto, ON, Canada; 4 Group Health Centre, Sault Ste, Marie, ON, Canada; 5 Department of Psychiatry, University of Pennsylvania and the Department of Veteran Affairs, Philadelphia, PA, USA; 6 25463 Waypoint Centre for Mental Health Care, Penetanguishene, ON, Canada

**Keywords:** depression, anxiety, alcohol use disorder, collaborative care, primary care, randomized clinical trial, computer-based assessment, measurement-based care, antidepressant, algorithm, treatment as usual

## Abstract

**Background:**

Collaborative care (CC) could improve outcomes in primary care patients with common mental conditions. We assessed the effectiveness of a transdiagnostic model of telephone-based CC (tCC) delivered by lay providers to primary care patients with depression, anxiety, or at-risk drinking.

**Methods:**

PARTNERS was a pragmatic trial in 502 primary care adults presenting with depressive symptoms, anxiety symptoms, or at-risk drinking randomized to (1) usual care by primary care providers (PCPs) enhanced with the results of computer-assisted telephone-based assessments (at baseline and 4, 8, and 12 months later) (enhanced usual care [eUC]) or (2) tCC consisting of eUC plus frequent telephone coaching and psychoeducation provided by mental health technicians who also communicated to the PCP recommendations from a psychiatrist for evidence-based pharmacotherapy, psychotherapy, or, when indicated, referrals to mental health services. The primary analysis compared the change on the 9-item Patient Health Questionnaire (PHQ-9) in participants presenting with depression (*n* = 366) randomized to tCC versus eUC. Secondary analyses compared changes on the Generalized Anxiety Disorder-7 scale (GAD-7) in those presenting with anxiety (*n* = 298); or change in the number of weekly drinks in those presenting with at-risk drinking (*n* = 176).

**Results:**

There were no treatment or time×treatment effects between tCC and eUC on PHQ-9 scores for patients with depression during the 12-month follow-up. However, there was a treatment effect (tCC > eUC) on GAD-7 scores in those with anxiety and a time×treatment interaction effect on the number of weekly drinks (tCC > eUC) in those with at-risk drinking.

**Conclusion:**

Implementing transdiagnostic tCC for common mental disorders using lay providers appears feasible in Canadian primary care. While tCC was not better than eUC for depression, there were some benefits for those with anxiety or at-risk drinking. Future studies will need to confirm whether tCC differentially benefits patients with depression, anxiety, or at-risk drinking.

## Abrégé

**Contexte:**

Les soins collaboratifs (SC) pourraient améliorer les résultats chez les patients des soins de première ligne souffrant d’états mentaux communs. Nous avons évalué l’efficacité d’un modèle transdiagnostique de SC par téléphone (SCt) dispensé par des prestataires non professionnels à des patients de première ligne souffrant de dépression, d’anxiété ou à risque de consommation d'alcool excessive.

**Méthodes:**

PARTNERS était un essai pragmatique mené auprès de 502 adultes recevants des soins de première ligne qui présentaient des symptômes dépressifs, d’anxiété ou une consommation à risque et qui ont été randomisés à : (1) des soins usuels par des prestataires de première ligne (PPL) améliorés par les résultats d’évaluations par téléphone assistées par ordinateur (au départ puis 4, 8, et 12 mois plus tard) (eSU); ou (2) SCt consistant en eSU et un fréquent encadrement par téléphone et psychoéducation donnés par des Techniciens de santé mentale (TSM) qui ont aussi communiqué aux PPL les recommandations d’un psychiatre à l’égard de la pharmacothérapie fondée sur des données probantes, de la psychothérapie ou, le cas échéant, des renvois aux services de santé mentale. La première analyse comparaît le changement du Questionnaire sur la santé du patient (QSP-9) chez les participants présentant une dépression (*n* = 366) randomisés aux SCt contre eSU. Les analyses secondaires comparaient le changement de l’échelle du Trouble anxieuxgénéralsé-7 (TAG-7) chez ceux présentant de l’anxiété (*n* = 298); ou le changement du nombre de consommations hebdomadaires pour ceux sujets à la consommation à risque (*n* = 176).

**Résultats:**

Il n’y avait pas d’effet de traitement ou d'interaction temps par traitement entre SCt et eSU aux scores QSP-9 pour les patients souffrant de dépression durant le suivi de 12 mois. Cependant, il y avait un effet de traitement (SCt > eSU) aux scores TAG-7 chez ceux souffrant d’anxiété et un effet d’interaction temps par traitement sur le nombre de consommations hebdomadaires (SCt > eSU) chez ceux à risque de boire.

**Conclusion:**

Mettre en œuvre des SCt transdiagnostiques pour les troubles mentaux communs avec l’aide de prestataires non professionnels semble faisable dans les soins de première ligne canadiens. Bien que SCt n’ait pas mieux réussi que eSU pour la dépression, il y avait certains bénéfices pour ceux souffrant d’anxiété ou à risque de consommation excessive. Les futures études devront confirmer si SCt bénéficiera différentiellement les patients souffrant de dépression, d’anxiété ou à risque de consommation excessive.

## Introduction

Depression, anxiety, and at-risk drinking are leading causes of disability worldwide.^1^ Up to a third of primary care patients experience symptoms of depression, anxiety, or at-risk alcohol use, although these conditions may be unrecognized by primary care providers (PCPs).^2^ Due to limited resources or time and competing clinical priorities, inadequate treatment is common despite the availability of effective pharmacotherapy or manual-based brief psychotherapy,^3–7^ leading to poor outcomes, increased health-care utilization, and a higher risk of suicide.^8–11^ The treatment of patients with comorbid disorders (e.g., depression and anxiety or alcohol misuse) can be particularly challenging.^5^ Limited face-to-face time impedes the systematic assessment of symptoms or adverse effects and the provision of psychoeducation, contributing to poor adherence and outcomes. ^6^

Collaborative care (CC) is a complex intervention based on the chronic disease management model.^7^ It has been shown to be efficacious in the treatment of depressive or anxiety disorder in primary care settings.^7^ A typical CC model consists of several components—case identification, baseline and follow-up assessments using standardized quantitative instruments (“measurement-based care (MBC)”) and treatment recommendations from a psychiatrist—that require intensive resources. Due to limited resources and a lack of access to mental health professionals, CC has not been widely adopted in primary care settings.^12,13–17^ To addresses these challenges in a Canadian context, we conducted a randomized clinical trial (RCT) that assessed the effectiveness of telephone-based CC (tCC) delivered by a mental health technician (MHT, a “lay provider”) to primary care patients with depression, anxiety, or at-risk drinking compared to enhanced usual care (eUC). We hypothesized that an intervention consisting of usual care plus tCC would be more effective in improving outcomes for primary care patients with depression, anxiety, or at-risk drinking, compared to usual care enhanced solely by measurement of symptoms (eUC).

## Methods

The methods of the study have been published^14^ and are summarized below. Primary care Assessment and Research of a Telephone intervention for Neuropsychiatric conditions with Education and Resources (PARTNERs) was a two-arm, double-blind, parallel RCT of a (remote) tCC intervention for primary care participants with depressive symptoms, anxiety symptoms, or at-risk drinking who were randomized to (1) eUC consisting of care provided by their PCP enhanced by the results of comprehensive computer-assisted telephone-based assessments (i.e., a form of MBC—see below) at baseline and 4, 8, and 12 months later; or (2) tCC consisting of eUC plus sessions provided by an MHT via phone, weekly during the first 2 to 4 months of the intervention and tapered gradually to monthly during the latter part of the intervention once participants improved. During these sessions, the MHT completed computer-assisted telephone monitoring of symptoms and adverse effects and offered psychoeducation and coaching. In addition, the MHT communicated to the PCP recommendations from a psychiatrist for evidence-based pharmacotherapy, psychotherapy, or, when indicated, for referrals to specialty mental health services.

All participants were recruited in primary care clinics in urban areas, suburban locations, and rural areas across Ontario (Canada) with all study activities conducted remotely by phone from a central office in Toronto. All participants signed an informed consent form approved by the Research Ethics Board at the Centre for Addiction and Mental Health, Toronto, Ontario. The trial was registered on ClinicalTrials.gov (Identifier: NCT02345122) on January 26, 2015.

The main inclusion criteria were age 18 years and older; receiving care at 1 of the participating clinics; self-referring or being referred by a PCP to the study because of depression, anxiety, or at-risk drinking; and ability to converse in English by telephone. The main exclusion criteria were lifetime diagnoses primarily reported by participants, including psychotic disorders, bipolar disorder, obsessive–compulsive disorder, or posttraumatic stress disorder; current substance use disorder other than alcohol use disorder; cognitive impairment with a score of 16 or above on the Blessed Orientation-Memory Concentration test (BOMC)^15^; high risk for suicide in the next year; or an unstable physical condition.

Research Associates (RAs) administered by telephone to all participants a comprehensive assessment at baseline, 4, 8, and 12 months later using the Behavioral Health Laboratory (BHL) software.^14^ This software has been widely used in the United States Veterans Administration system; it facilitates adaptive interviews based on validated standardized instruments, including the 9-item Patient Health Questionnaire (PHQ-9),^16^ 7-item Generalized Anxiety Disorder scale (GAD-7),^17^ the assessment of the self-reported number of drinks per week and binges during the past 3 months, Veterans RAND 12-Item Health Survey (VR-12),^18^ and BOMC. In addition, the RAs had access to a standard operating procedure to assess and manage suicidality. After study completion, the RAs reviewed clinical charts to abstract the documentation of interventions (e.g., medications and dosages) participants received while they were in the study.

Participants assessed at baseline to have depression (defined as a PHQ-9 score ≥10), anxiety (defined as a GAD-7 score ≥10), or at-risk drinking (exceeding the Canadian Guidelines for safe drinking, which are based on the number of drinks or binges^19^) were randomized stratifying for the participant's site and based on the presenting condition(s). The participants and the RAs performing the baseline and follow-up assessments were blind to the randomization; by design, the PCP and the tCC team (i.e., the study psychiatrist and MHTs) were informed of the randomization.

Participants randomized to tCC were assigned to a trained bachelor-level MHT (i.e., a “lay provider”^20–25^) who completed additional computer-assisted assessments and reviewed all available information with the study psychiatrist (DJR) during weekly group supervision meetings. The study psychiatrist made a formal treatment plan guided by a flowchart treatment algorithm^14^ and the MHT e-faxed recommendations to the PCP who was free to implement them or not or to provide alternative care as they saw fit.

During the initial weekly, then bi-monthly, and finally monthly phone calls, MHTs also helped CC participants to identify resources (e.g., apps and community-based services) and provided monitoring including the administration of the PHQ-9, GAD-7, or an inquiry about the number of drinks, as relevant. MHTs also monitored treatment adherence and adverse effects and provided continuing psychoeducation related to the participant's conditions and their treatment.

The RAs assessed the participants randomized to eUC by phone with the same BHL software at the same time points. The results of these symptom assessments were e-faxed to PCPs, but no recommendations were provided. PCPs managed these participants as they saw fit. To maintain the blind, eUC participants were also contacted by an MHT after the completion of each assessment, and they were briefly informed about the results of their assessment and told that they would be contacted again in 4 months.

The primary outcome measure was the change in PHQ-9 from baseline to 12 months in those presenting with depression (i.e., those with a PHQ-9 ≥10 at baseline). Because of the transdiagnostic recruitment strategy, preplanned secondary outcome measures included change in GAD-7 from baseline to 12 months in those presenting with anxiety (i.e., those with a GAD-7 score ≥10 at baseline); change in the number of weekly drinks from baseline to 12 months in those presenting with at-risk drinking (i.e., those exceeding the Canadian Guidelines for safe drinking at baseline); and the documentation of initiation and adequacy of antidepressant treatment using the Antidepressant Treatment History Form^26^ in participants whose health records were reviewed. We also analyzed the rates of initiation or change in antidepressant medications or psychosocial interventions reported by participants in the whole sample.

Additional preplanned outcomes included change in health-related quality of life and functioning in the whole sample as measured by the VR-12; rates of response or remission for depression in the depression group (defined as PHQ-9 score <10 and <5, respectively) or for anxiety in the anxiety group (defined as GAD-7 score <10 and <5, respectively), and decrease in the number of weekly drinks to meeting the guidelines for safe drinking.

The main analyses compared changes from baseline to 12 months between the 2 treatment groups with linear contrasts of estimated means for the 3 main outcome measures (i.e., PHQ-9, GAD-7, and number of drinks). Accounting for the clustering within subjects and sites, mixed models assessed changes in depression or anxiety scores and changes in health-related quality of life and functioning. Because of skewed distribution, a generalized estimating equation (GEE) assessed changes in the number of drinks. Both mixed models and GEE were specified with a time-by-group interaction, where time was considered categorical. Given the high rates of comorbidity, the 3 models adjusted for baseline PHQ-9 scores, GAD-7 scores, and number of drinks. A similar approach was used for the analysis of changes in VR-12 scores. Logistic regressions compared rates of remission, response, or safe drinking at 12 months in the 2 treatment groups. We used multiple imputation to impute missing values for response and remission at 12 months. Remission and response were the dependent variable of our logistic regression to look at treatment-group differences for these outcomes. A logistic regression assessed whether the proportion of participants reporting receiving treatment differed in the 2 treatment groups. Rates of initiation and adequacy of antidepressant treatment documented in health records were compared with Fisher tests. The analyses of response and remission rates were conducted using Mplus, and all other analyses using SAS System 9.4.

We had planned to recruit a sample of 1,000 participants that should have provided enough power to detect any clinically meaningful effect of the intervention. Since we were able to randomize only half of this planned sample and a post-hoc power calculation is not statistically sound, we estimated the detectable effect size, given the size of our sample and the characteristics of our model. Considering 80% power and significance level of 0.05, the detectable effect size was a difference between treatment groups in changes from baseline to 12 months of 1.85 for the PHQ-9, 1.90 for the GAD-7, and 5.7 drinks per week. This calculation was conducted using our data and a series of 1,000 Monte Carlo simulations for each outcome with the R package SIMR.^27^ Since the package does not accommodate GEE models, the calculation for the number of drinks was approximated by a normal mixed model in the original scale.

## Results

**Figure 1.**
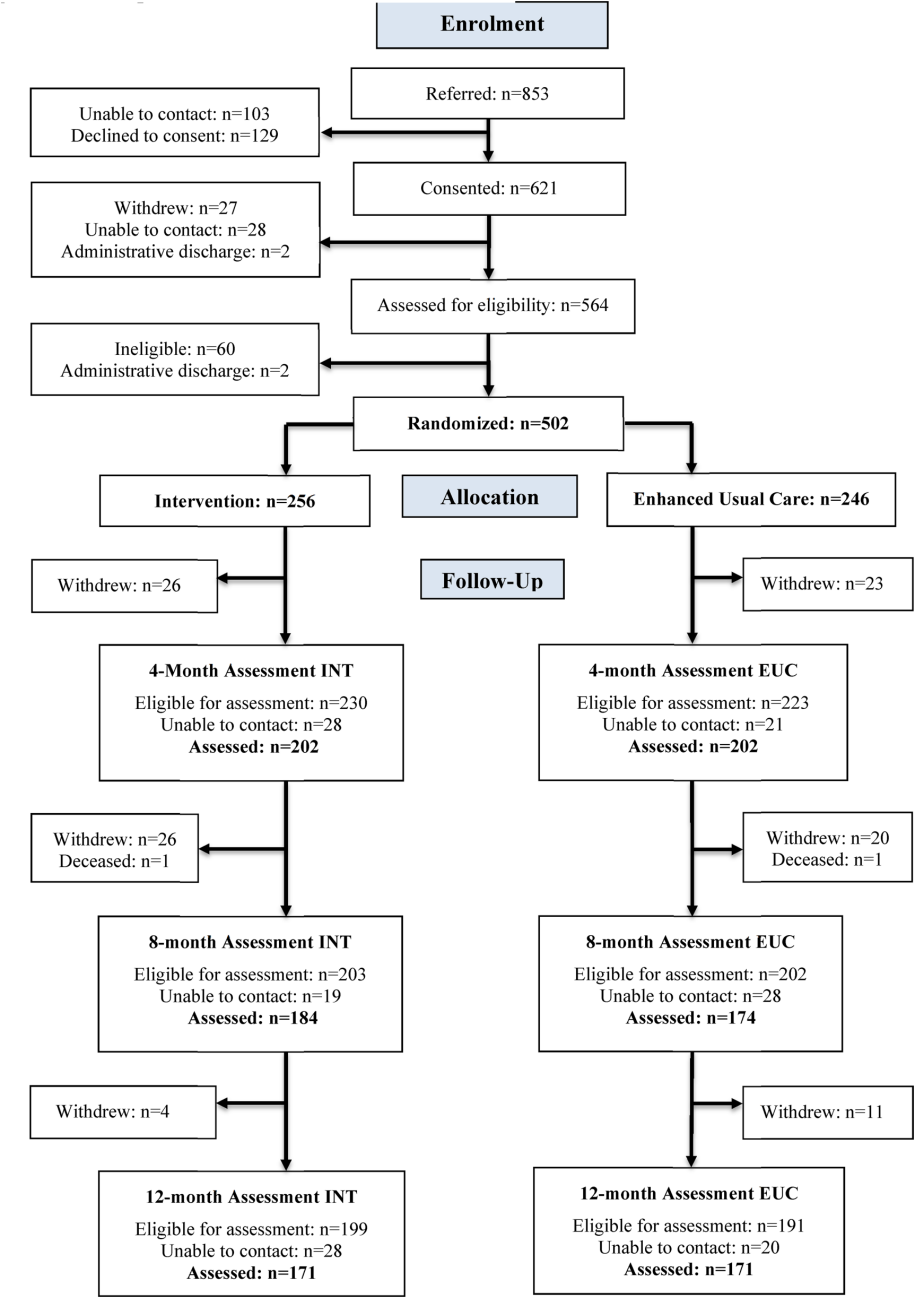
Participant flow in the PARTNERs study.

From November 2014 to April 2018, we randomized 502 participants. Figure 1 summarizes participant flow through the trial. At 12 months, 171 of 256 (66.8%) participants in the tCC group and 171 of 246 (69.5%) in the eUC group completed the evaluation (Figure 1), reflecting similar rates of drop-out due to a formal withdrawal of consent or our inability to contact the participants. Seven serious adverse events (SAEs) in the tCC group comprised 1 death due to a myocardial infarction, 4 SAEs related to suicidality, 1 threat to hurt somebody, and 1 unrelated physical problem; 5 SAEs in the eUC group comprised 1 death of unknown cause, 1 SAE related to suicidality, 2 related to alcohol misuse, and 1 unrelated physical problem.

**Figure 2.**
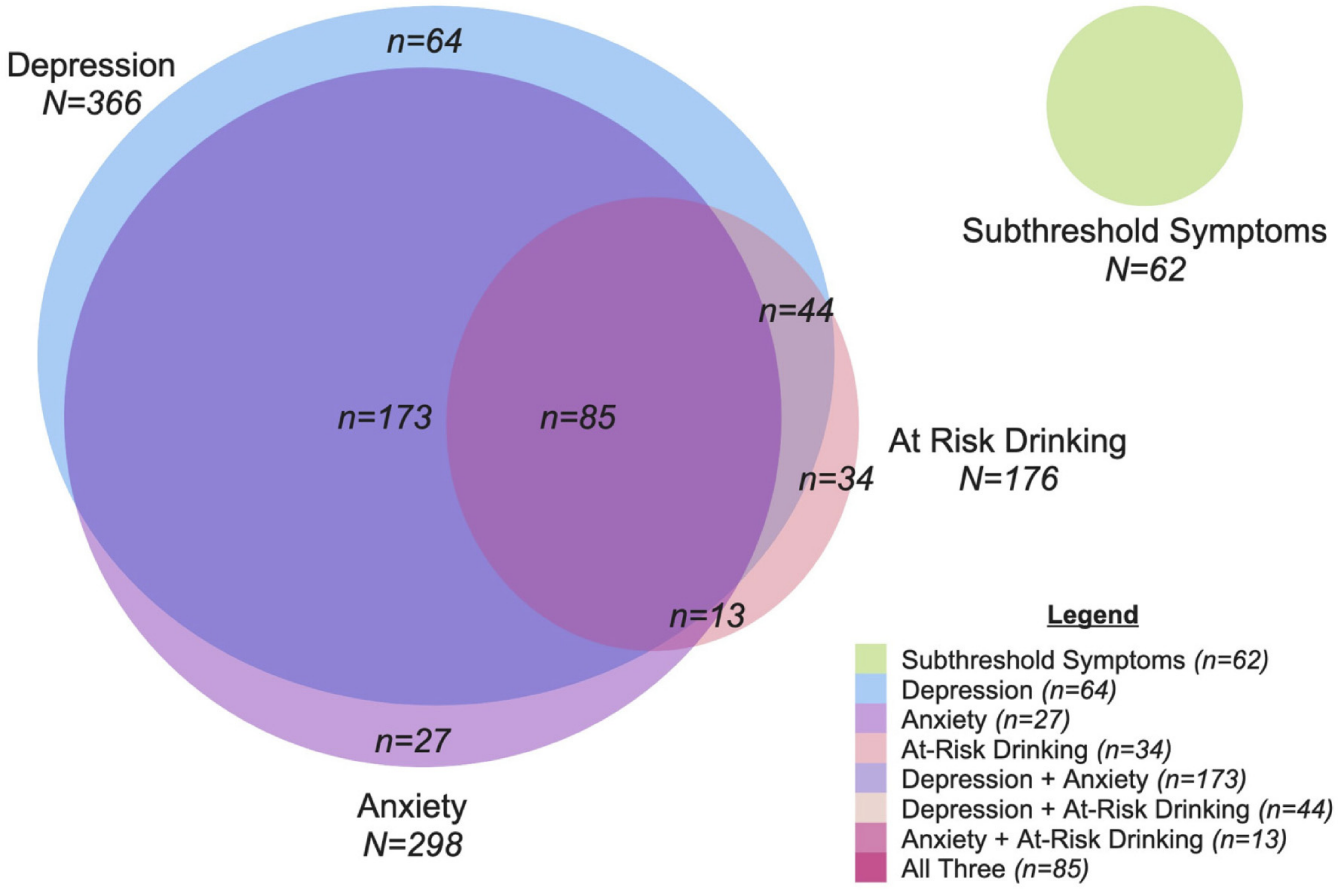
Distribution of participants in diagnostic groups in the PARTNERs study. See text for definitions of diagnostic groups.

Table 1 summarizes the participant characteristics, overall and in the 2 treatment groups. None of these characteristics differed significantly between the 2 treatment groups at baseline (analysis not shown). Figure 2 shows the distribution of participants in the 4 diagnostic groups (i.e., depression, anxiety, at-risk drinking, and subthreshold symptoms) and their overlap; only 125 (24.9%) of the 502 participants belonged solely to 1 diagnostic group, with 85 (16.9%) belonging to all 3. More than two-thirds of participants (*n* = 343; 68.3%) reported taking a psychotropic medication in the month prior to the baseline visit.

**Table 1.** Baseline Characteristics of Randomized Participants in PARTNERs.

	Whole sample (*N* = 502)	Collaborative care group (*n* = 256)	Enhanced usual care group (*n* = 246)
Age (years)	41.3 ± 15.7 (18–90)	41.5 ± 15.5 (18–81)	41.0 ± 15.9 (18–90)
Self-reported sex at birth Female Male	334 (66.5%) 168 (33.5%)	166 (64.8%) 90 (35.2%)	168 (68.3%) 78 (31.7%)
Self-reported race - White - Black - Asian - Indigenous - Other/mixed - Preferred not to answer	389 (77.5%) 13 (2.6%) 47 (9.4%) 17 (3.4%) 35 (7.0%) 1 (0.2%)	195 (76.2%) 7 (2.7%) 21 (8.2%) 11 (4.3%) 21 (8.2%) 1 (0.4%)	194 (78.9%) 6 (2.4%) 26 (10.6%) 6 (2.4%) 14 (5.7%) 0 (0.0%)
Marital status - Never married - Married/partnered - Divorced/separated - Widowed	203 (40.4%) 219 (43.6%) 61 (12.2%) 19 (3.8%)	102 (39.8%) 109 (42.6%) 34 (13.3%) 11 (4.3%)	101 (41.1%) 110 (44.7%) 27 (11.0%) 8 (3.3%)
Work status* - Working full-time - Working part-time - Not working	191 (38.0%) 82 (16.3%) 229 (45.6%)	99 (38.7%) 42 (16.4%) 115 (44.9%)	92 (37.4%) 40 (16.3%) 114 (46.3%)
Financial status - Struggling financially - Enough to get along - Comfortable	62 (12.4%) 200 (39.8%) 240 (47.8%)	33 (12.9%) 105 (41.0%) 118 (46.1%)	29 (11.8%) 95 (38.6%) 122 (49.6%)
Self-rated health - Excellent/very good - Good - Fair - Poor	132 (26.3%) 222 (44.2%) 116 (23.1%) 32 (6.4%)	73 (28.5%) 112 (43.8%) 58 (22.7%) 13 (5.1%)	59 (24.0%) 110 (44.7%) 58 (23.6%) 19 (7.7%)
Chronic pain	232 (46.2%)	125 (48.8%)	107 (43.5%)
Current smoker	111 (22.1%)	65 (25.4%)	46 (18.7%)
Condition identified on referral - None - Depression (PHQ-9 ≥10) - Anxiety (GAD-7 ≥10) - At-risk drinking - Depression and anxiety - Depression and at-risk drinking - Anxiety and at-risk drinking - All 3 condition	62 (12.4%) 64 (12.7%) 27 (5.4%) 34 (6.8%) 173 (34.5%) 44 (8.8%) 13 (2.6%) 85 (16.9%)	31 (12.1%) 33 (12.9%) 12 (4.7%) 19 (7.4%) 85 (33.2%) 22 (8.6%) 7 (2.7%) 47 (18.4%)	31 (12.6%) 31 (12.6%) 15 (6.1%) 15 (6.1%) 88 (35.8%) 22 (8.9%) 6 (2.4%) 38 (15.4%)
- PHQ-9	13.3 ± 5.8 (1–27)	13.2 ± 5.7 (1–26)	13.5 ± 5.9 (2–27)
- GAD-7	11.0 ± 5.4 (0–21)	11.0 ± 5.6 (0–21)	11.0 ± 5.2 (0–21)
- Number of drinks per week**	6.9 ± 12.9 (0–113)	7.0 ± 13.7 (0–113)	6.8 ± 12.0 (0–70)

All data are presented as mean ± SD (range) or *n* (%).

GAD-7: 7-item Generalized Anxiety Disorder scale; PHQ-9: 9-item Patient Health Questionnaire.

*Includes 68 students in the whole group (35 in the collaborative care group and 33 in the enhanced usual care group).

**Median number of drinks per week: 2.0 in the whole group; 1.0 in the collaborative care group; and 2.0 in the enhanced usual care group.

### Primary and Secondary Outcomes in Each Diagnostic Group

**Figure 3.**
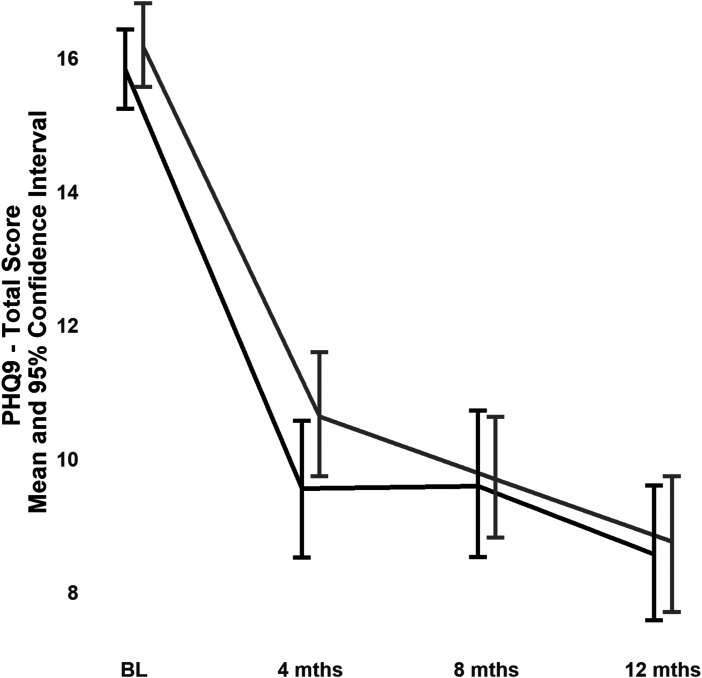
PHQ-9 scores in the 366 participants with PHQ-9 ≥10 at baseline (187 in the telephone collaborative care group and 179 in the enhanced usual care group).

*Note*. Collaborative care group: black line; enhanced usual care group: grey line. BL: baseline; mths: months; PHQ-9: 9-item Patient Health Questionnaire.

For the primary clinical outcome (i.e., change in PHQ-9 scores in participants with a PHQ-9 score of ≥10 at baseline), the test for the contrast between the two treatment groups was not significant (mean (SE) difference between treatment groups in change in PHQ-9 score from baseline to 12 months: −0.19 (0.66); *t*(910) =−0.29, *P* = 0.77). The estimated marginal means plot showed minimal difference in the trajectories between groups (Figure 3). There was a significant time effect, *F*(3,887) = 209, *P* < 0.001, indicating that both groups improved over time but there was no treatment by time interaction, *F*(3,887) = 1.20, *P* = 0.31.

**Figure 4.**
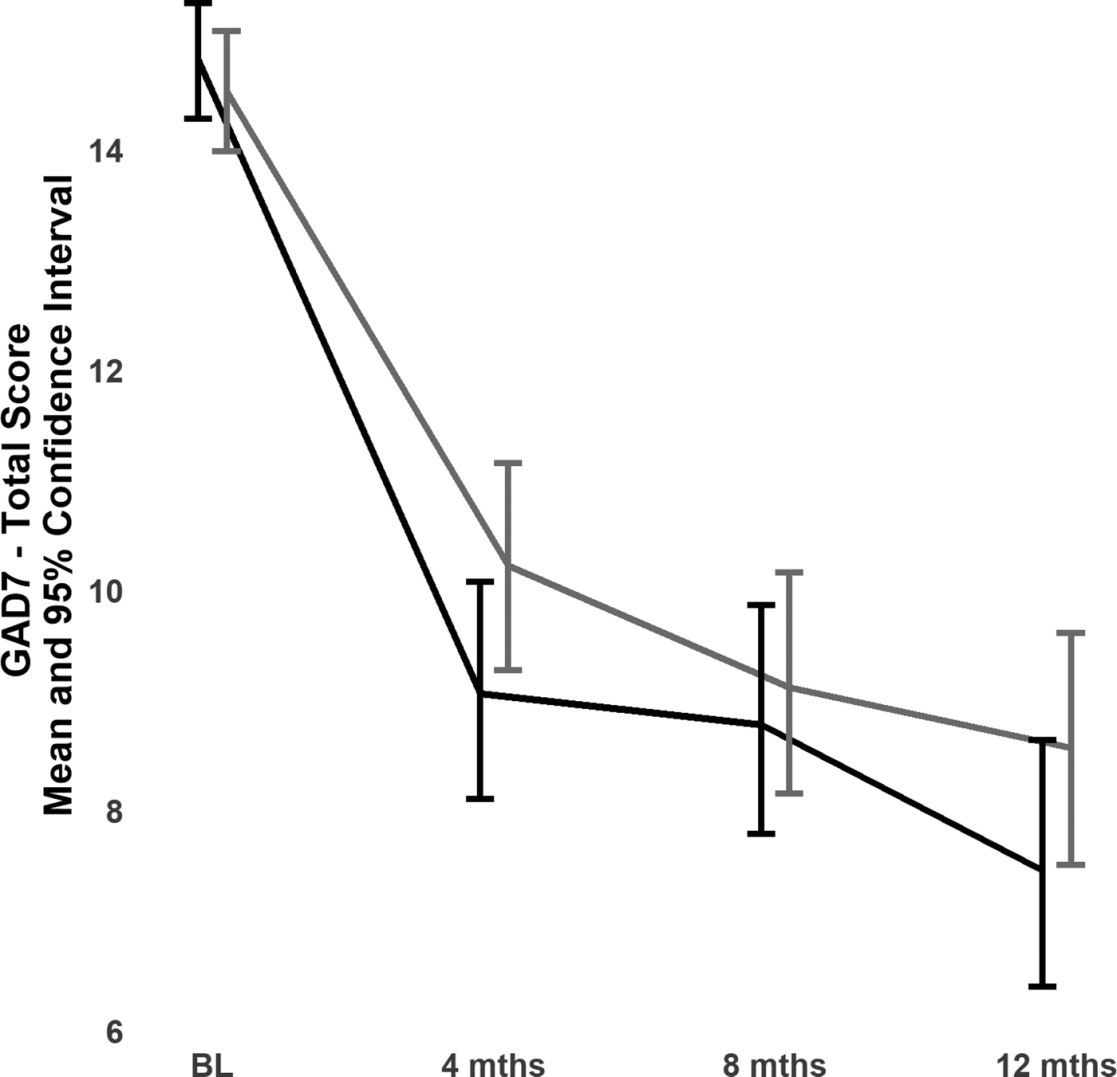
GAD-7 scores in the 298 participants with GAD-7 ≥10 at baseline (151 in the telephone collaborative care group and 147 in the enhanced usual care group).

*Note*. Collaborative Care group: black line; enhanced usual care group: grey line. BL: baseline; mths: months; GAD-7: 7-item Generalized Anxiety Disorder scale.

For a change in GAD-7 in participants with a GAD-7 score of ≥10 at baseline, the test for the contrast between the 2 treatment groups was significant (mean (standard error, SE) difference in change in GAD-7 score from baseline to 12 months: 1.47 (0.67), *t*(733) = 2.19, *P* = 0.029), with a larger decrease in GAD-7 scores in the tCC group than in the eUC group (Figure 4).

**Figure 5.**
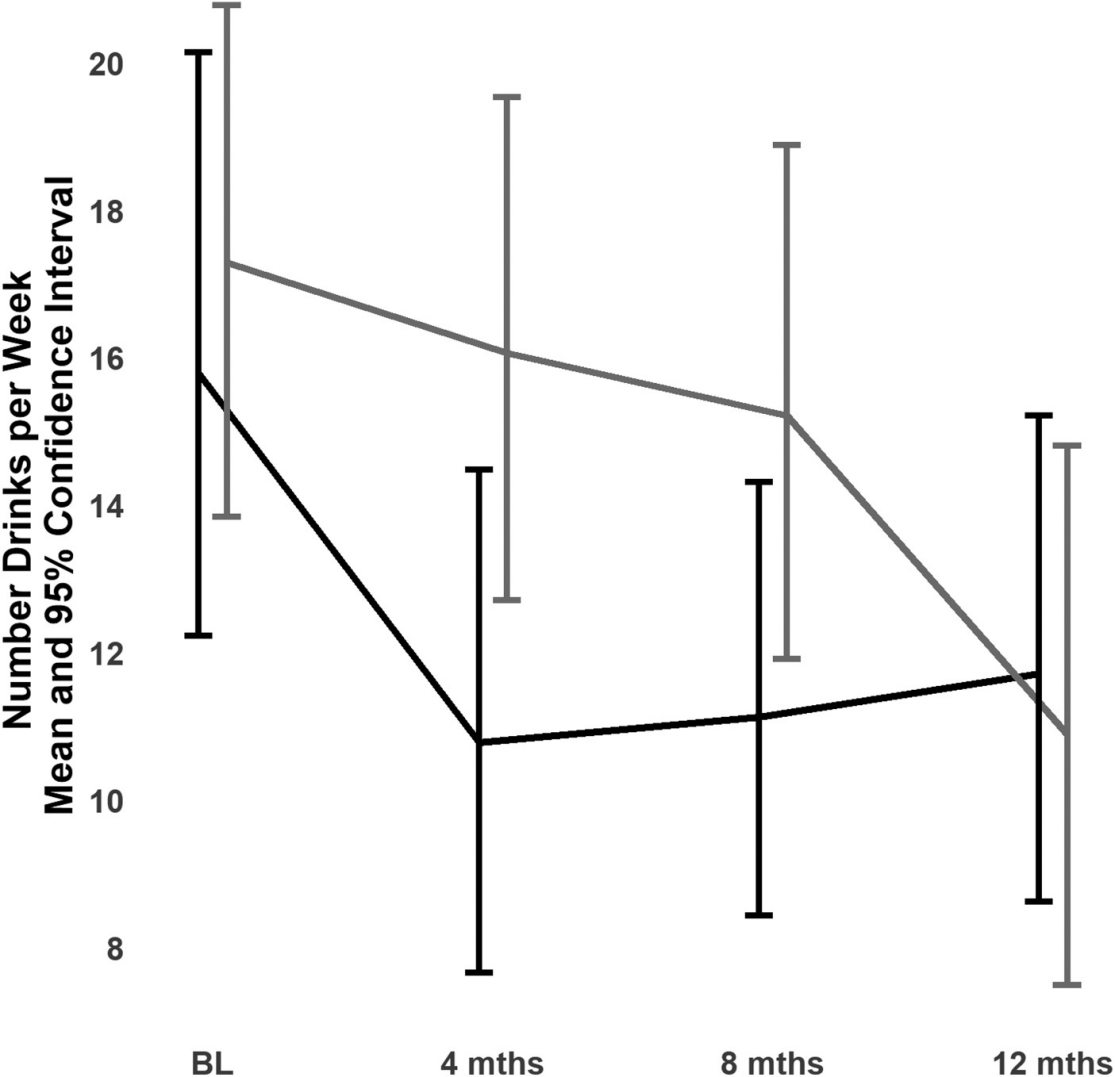
Number of weekly drinks in the 176 participants with at-risk drinking at baseline (95 in the telephone collaborative care group and 81 in the enhanced usual care group).

*Note*. Collaborative care group: black line; enhanced usual care group: grey line. BL: baseline; mths: months.

For the number of weekly drinks in participants with at-risk drinking, the contrast was not significant (ratio of per cent changes in the number of daily drinks from baseline to 12 months: 0.89 (95% CI, 0.63 to 1.27), t(368) = −0.63, *P* = 0.53). Hence, there was no treatment effect: i.e., after 12 months, participants with at-risk drinking in the tCC and eUC groups did not differ in their number of weekly drinks. However, the tCC and eUC groups had different trajectories (significant treatment × time interaction: *F*(3,368) = 2.68, *P* = 0.047), with the tCC group improving significantly more than the eUC group after 4 months (ratio of per cent changes in the number of daily drinks from baseline to 4 months: 1.63, 95% CI, 1.07 to 1.92, *t*(368) = 2.39, *P* = 0.017) but not by the end of 8 months (ratio of per cent changes: 1.25, 95% CI, 0.90 to 1.74, *t*(368) = 1.33, *P* = 0.185) (Figure 5).

Estimates of the effect sizes of the continuous measures were: PHQ-9: Cohen's d = 0.057; 95% CI, −0.29 to 0.404; GAD-7: Cohen's d = 0.38; 95% CI, −0.036 to 0.795; number of weekly drinks: Cohen's d = −0.099; 95% CI, −0.352 to 0.154.

### Initiation or Change in Antidepressant Treatment

The review of health records showed higher rates of documentation of initiation of antidepressants (Fisher's *P* = 0.004) and of adequate antidepressant trials (Fisher's *P* = 0.007) in the tCC group than in the eUC group. Similarly, in the whole sample, compared to eUC participants, tCC participants were significantly more likely to report initiation or change in antidepressant (*t*(1094) = −3.15; *P* = 0.002), and these changes occurred earlier (group by time interaction (*F*(3,1094) = 3.60, *P* = 0.013). There were no significant differences in initiation or change of psychosocial interventions in the whole sample (*t*(1093) = −0.54, *P* = 0.59), or in any of the diagnostic subgroups (data not shown). Models including the study site showed no significant site effect or site as moderator of group effect.

### Quality of Life and Functioning in the Whole Sample

There was a significant time effect for VR-12 mental scores (*F*(3,425) = 86.17, *P* < 0.001), and the VR-12 physical scores (*F*(3,306)= 3.54, *P* = 0.0151), showing an increase in VR-12 mental scores and a decrease in VR-12 physical scores over time. However, when looking at treatment × time interactions, no differences were found between the tCC and eUC groups in VR-12 mental scores trajectories from baseline to 12 months (*F*(3,1206) = 0.04, *P* = 0.99), or in their changes from baseline to 12 months (*t*(1501) = −0.13, *P* = 0.90); similarly, no differences were found in VR-12 physical scores trajectories (*F*(3,1182) = 0.33, *P* = 0.80) or in their changes from baseline to 12 months (*t*(1513) = 0.58, *P* = 0.56).

### Response and Remission Rates

Inferential results related to logistic regression modelling of response and remission rates are presented in Table 2. Using the planned definitions of response (“response 1”; see above) and remission, there were no significant differences between the response and remission rates between the tCC and eUC groups for any of the 3 diagnostic groups. At the request of a reviewer, we also conducted a post-hoc analysis defining response more traditionally as >50% reduction in PHQ-9 scores, GAD-7 scores, or number of weekly drinks (“response 2”). In this analysis, the odds of responding were significantly higher in the tCC group than the eUC group for those presenting with anxiety but did not differ significantly between the tCC and eUC groups for participants presenting with depression or at-risk drinking (see Table 3).

**Table 2.** Rates (Denominator/Numerator) of Response or Remission for Depression, or Anxiety, and Resolution of At-Risk Drinking.

	Telephone collaborative care group	Enhanced usual care group
Depression		
Response 1	62.6% (77/123)	61.8% (76/123)
Response 2	53.7% (66/123)	50.4% (62/123)
Remission	30.1% (37/123)	24.4% (30/123)
Anxiety		
Response 1	71.1% (69/97)	59.8% (58/97)
Response 2	63.9% (62/97)	43.2% (42/97)
Remission	38.1% (37/97)	23.7% (23/97)
At-risk drinking		
Response 2	40.0% (22/55)	42.2% (19/45)
Safe drinking	8.3% (5/60)	3.8% (2/53)

**Table 3.** Comparison of Rates of Response or Remission for Depression, or Anxiety, and Resolution of At-Risk Drinking.

Outcome	Odds ratio*	Lower 95% CI	Upper 95% CI	*t*-value**	*P*-value
Depression					
Response 1	0.99	0.60	1.63	−0.03	0.972
Response 2	0.86	0.54	1.38	−0.62	0.539
Remission	0.82	0.51	1.32	−0.811	0.418
Anxiety					
Response 1	0.62	0.33	1.15	−1.51	0.131
Response 2	0.52	0.28	0.94	−2.17	0.030
Remission	0.58	0.30	1.10	−1.67	0.094
At-risk drinking					
Response 2	0.90	0.48	11.69	−0.330	0.741
Safe drinking	0.63	0.22	1.84	−0.85	0.396

See text for definitions of depression, anxiety, at-risk drinking, remission, response, and safe drinking.

*Odds of the outcome event in the enhanced usual care group divided by the odds in the collaborative care group: an odds ratio below 1 indicated more events happening in the collaborative care group.

**This statistic is calculated as the log odds divided by its standard error and the *P*-value is approximated using the normal distribution.

## Discussion

In this RCT of over 500 primary care patients with symptoms of depression, anxiety, or at-risk drinking, compared with eUC, a novel, low-cost, transdiagnostic tCC intervention delivered by lay providers did not result in a larger improvement in depression symptoms in those presenting with depression at baseline or in health-related quality of life and functioning in the whole sample. In those presenting with anxiety, tCC yielded a larger improvement in anxiety symptoms. While tCC did not affect the proportion of participants who had transitioned from at-risk drinking to safe drinking at the end of the 12-month study, it was associated with a faster transition. Participants randomized to tCC were more likely to have antidepressants initiated and to receive adequate antidepressant treatment than those randomized to eUC. These findings suggest that tCC delivered by lay providers is feasible in primary care and may benefit some patients with anxiety or at-risk drinking. These findings are also relevant in the context of the COVID-19 pandemic, which has led to a higher prevalence of anxiety symptoms and substance misuse and a rapid increase in virtual health care.^28,29^

Improvement in depressive symptoms and rates of depression response or remission were high in both groups; contrary to our hypotheses, they were not significantly different in the tCC or eUC groups. This is despite the fact that those receiving tCC were more likely to be prescribed an adequate antidepressant trial. This finding is inconsistent with similar previous studies.^30^ One possible explanation is the high rates of depression response observed with both tCC and eUC. These rates are surprising given the selective nature of the referrals to the study, with a mean (standard deviation, SD) of only 0.3 (0.7) referrals per month per PCP (and a handful of self-referrals) suggesting that PCP selectively referred patients who were “hard to treat.”^14^ Satisfaction data of participants and qualitative interviews of PCPs showed that participants and the PCPs valued both tCC and eUC, suggesting that they found both to be beneficial.^31,32^

The positive effect of our active intervention on anxiety symptoms is consistent with a previous trial of a telephone-based tCC for 329 primary care patients with anxiety^33^ or a study of computerized cognitive behavioural therapy (cCBT) with telephone support from lay providers.^34^ In a more recent trial, online CC (in the form of an internet support group) provided no additional benefit for improving mood and anxiety symptoms in primary care over guided cCBT, but guided cCBT was more effective than usual care.^35^

Similarly, in a recent large RCT in primary care patients with depression or anxiety, sertraline was superior to placebo for anxiety symptoms but not depressive symptoms.^34^ Taken together with our results, this suggests that anxiety symptoms are more likely to require specific treatment than depressive symptoms in primary care patients—i.e., they are less likely to improve with the passing of time and nonspecific intervention, such as symptom monitoring, which account for approximately 80% of improvement in depressive symptoms in a typical study.^36^ By contrast, MBC, consisting of usual care enhanced with structured symptom assessment (as in our eUC group), may be sufficient to produce clinical improvement in many patients with depressive symptoms.^37^ MBC may enhance treatment outcomes by updating clinicians about their patient's symptoms, allowing for tailored therapies. MBC can also improve patients’ engagement and treatment adherence.^37^

Our results also add to the limited literature on CC interventions for alcohol use disorder. Two published RCTs of (face-to-face) CC for alcohol use disorder have reported conflicting results.^38,39^ To our knowledge there are no other RCTs of CC, delivered face-to-face, via telephone, or otherwise, for at-risk drinking in primary care.

Our study has both strengths and limitations. Although we only randomized half of the planned sample, the relatively large sample size and high retention rates (68.5% overall) provided sufficient power to detect clinically meaningful differences. Selecting participants based on referrals from their PCP and their scores on the PHQ-9 or GAD-7 rather than formal psychiatric diagnoses has some pragmatic advantage (i.e., it better reflects primary care practice). However, it may have resulted in the inclusion of participants with adjustment disorders or subsyndromal depression, both of which have been shown to resolve with the passing of time or placebo treatment,^40^ contributing to our negative findings. Randomizing participants instead of randomizing PCPs or primary care practices could have led to “contamination”: taking care of participants in the tCC group may have helped PCPs to manage their patients in the eUC group (e.g., by using the recommended antidepressants and dosages). However, the adequacy of antidepressant treatment was higher in the tCC group. Moreover, while contamination can occur in depression RCTs, it has had minimal impact in a previous depression RCT in primary care,^41^ and an RCT of CC for primary care depression has shown results similar to our study, despite using cluster randomization.^42^ Another limitation of our study is the potential lack of generalizability of our findings. As mentioned above, PCPs were very selective in the patients they referred to the study.^14^ Also, while we made efforts to recruit various types of practices (e.g., family health teams and solo PCPs) in locations throughout Ontario, these practices may not be representative of all primary care practices in Ontario and even less so in other jurisdictions.

In conclusion, the findings of our RCT, taken together with the results of other CC trials, support the feasibility of implementing transdiagnostic tCC delivered by lay providers in primary care. However, while patients with anxiety or at-risk drinking benefitted from tCC compared to eUC, this was not the case for those with depression. This negative result goes against our hypothesis that the crucial “ingredient” of the active intervention would be the MHT. Given the marked improvement in depressive symptoms with both tCC and eUC, future studies of CC for depression need to assess the benefits and cost-effectiveness of its specific components, in particular, MBC.

## Supplemental Material

sj-docx-1-cpa-10.1177_07067437231156243 - Supplemental material for A Collaborative-Care Telephone-Based Intervention for Depression, Anxiety, and at-Risk Drinking in Primary Care: The PARTNERs Randomized Clinical Trial Click here for additional data file. Supplemental material, sj-docx-1-cpa-10.1177_07067437231156243 for A Collaborative-Care Telephone-Based Intervention for Depression, Anxiety, and at-Risk Drinking in Primary Care: The PARTNERs Randomized Clinical Trial by M. Ishrat Husain, David J. Rodie, Athina Perivolaris, Marcos Sanches, Allison Crawford, Kyle P. Fitzgibbon, Andrea Levinson, Rose Geist, Paul Kurdyak, Brian Mitchell, David Oslin, Nadiya Sunderji, Benoit H. Mulsant and in The Canadian Journal of Psychiatry
