# Supplementary material for: A Collaborative-Care Telephone-Based Intervention for Depression, Anxiety, and at-Risk Drinking in Primary Care: The PARTNERs Randomized Clinical Trial
Source: Can J Psychiatry. 2023 Feb 28;68(10):732–44. doi: 10.1177/07067437231156243 (PMC10517649; doi:10.1177/07067437231156243)
Supplement: sj-docx-1-cpa-10.1177_07067437231156243 - Supplemental material for A Collaborative-Care Telephone-Based Intervention for Depression, Anxiety, and at-Risk Drinking in Primary Care: The PARTNERs Randomized Clinical Trial [file sj-docx-1-cpa-10.1177_07067437231156243.docx]

**SUPPLEMENTATRY DESCRIPTION OF METHODS**

**Overall Study Design**

The methods for the Primary Care Assessment and Research of a Telephone Intervention for Neuropsychiatric Conditions with Education and Resources (PARTNERs) study have been described in details in three previous manuscripts.^1-3^ In brief, PARTNERs was a two-arm, blinded, parallel randomized controlled trial (RCT) comparing the effectiveness of a telephone collaborative care (tCC) intervention to improve depressive symptoms, anxiety symptoms, or at-risk drinking in primary care patients. Eligible referred patients were randomized to one of two conditions: (1) enhanced usual care (eUC) consisting of care provided by their primary care providers (PCP) enhanced by the result of assessments at baseline and 4, 8, and 12 months later; or (2) tCC consisting of: eUC plus: treatment recommendations from the study psychiatrist for evidence-based pharmacotherapy to be prescribed by the PCP, psychotherapy, or, when indicated, recommendations for referrals to specialty mental health services; telephone-based computer-assisted regular monitoring of symptoms, and adverse effects; and telephone-based psychoeducation and coaching provided by a Mental Health Technician (MHT). These telephone meetings between the MHT and the participants took place weekly during the first few months of participation, then once the participant’s condition improved every two weeks and them monthly. The MHTs participated in weekly group supervision provided by the study psychiatrist.

**Study Setting and Participants**

All participants were recruited in Ontario, Canada; they were patients in primary care team-based organizations, a University-based primary care clinic, a clinic led by NPs, and offices of solo PCPs. The clinical sites were distributed in urban areas, suburban locations, and rural areas with all study activities conducted by phone from a central office in Toronto.^1^

**Inclusion criteria** were: age 18 years and older; receiving care from a PCP participating in the study; Patient Health Questionnaire (PHQ-9)^4^ score > 10, 7-item Generalized Anxiety Disorder scale score (GAD-7)^5^ > 10, or alcohol use exceeding the Canadian Guidelines^6^ (see below); having access to a telephone; corrected auditory acuity that enables conversation in English by telephone; and willingness and ability to provide written informed consent. **Exclusion criteria** were: lifetime primary psychotic illness, bipolar disorder, obsessive-compulsive disorder, or posttraumatic stress disorder; current substance use disorder other than alcohol use disorder; clinically significant cognitive impairment (as indicated by a score of 16 or above on the Blessed Orientation Memory Concentration Test (BOMC)7; high risk for suicide in the next year based on the 5-item Paykel’s questionnaire^8^ and the opinion of the study psychiatrist; physical instability as evidenced by current hospitalization; and being expected by one’s PCP to die during the next 6 months.

Potential participants were asked whether they had been diagnosed with the exclusionary diagnoses listed above. In addition, some “exclusionary diagnoses” were determined based on cut-off scores (e.g., the PTSD Checklist for DSM-5; PCL-5^9^). Finally, when the presence of an “exclusionary diagnosis” was unclear (e.g., bipolar disorder vs. major depressive disorder (MDD)), the RA consulted with the study psychiatrist and, on occasion, the study psychiatrist interviewed the potential participant to clarify diagnosis.

**Referral and Recruitment**

Patients were identified and referred by participating PCPs or a member of the care team (e.g., a nurse or a social worker) as suffering from clinically significant depression, anxiety, or at-risk drinking (including alcohol use disorder) that could benefit from further assessment and treatment. Information cards were also placed in the waiting rooms and offices of some of the participating clinics and patients could self-refer by calling the research staff directly.

The study was conducted in accordance with the principles of the Declaration of Helsinki and approved by the Research Ethics Board at the Centre for Addiction and Mental Health, Toronto, Ontario. The trial was registered on ClinicalTrials.gov (NCT02345122) on January 26, 2015.

**Assessments and Data Collection**

All assessments were conducted via telephone by trained bachelor-level Research Associates (RAs). All participants received a baseline assessment and follow-up assessments after 4, 8 and 12 months. The RAs remained blinded to allocation throughout the duration of the study. Additionally, after the completion of the one-year observation period, all the participants’ clinical charts were reviewed to abstract the intervention (e.g., medications, doses, refills) and other health services (e.g., laboratory tests, referrals, mental health evaluations) participants received while they were in the study. Collection, storage and access of these data were done using a REDCap interface and database.^10^

The specialized Behavioral Health Laboratory (BHL) software developed by CAPITAL SOLUTION DESIGN in partnership with the MIRECC VISN4 Mental Illness Research, Education and Clinical Center at the University of Pennsylvania for the Department of Veteran’s Affairs (VA) was used to collect, store, and access the baseline, 4-, 8-, and 12-month assessment data. This software has been widely used in the US VA system; it facilitates adaptive interviews based on validated standardized instruments.^11^ In addition, the RAs had access to a standard operating procedure to assess and manage suicidality.

**Randomization and Allocation Concealment**

Randomization was at the level of the participant (controlling for the participant’s site) to allow for stratification based on the presenting condition(s) identified during the baseline assessment and flagged by the BHL software. Depression was defined by as score of 10 or higher on the PHQ-9; anxiety by a score of 10 or higher on the GAD-7, and at-risk drinking (including alcohol use disorder) defined as drinking 10 or more drinks per week for a female, 15 or more drinks per week for a male, or having 4 or more binges during the past 3 months - with a binge defined as 4 or more drinks in a single day for a female and 5 or more drinks in a single day for a male.

The study biostatistician prepared the randomization schedule with randomization stratified by site and the seven possible combinations of the three presenting conditions (i.e., depression, anxiety, at-risk drinking, depression + anxiety, depression + at-risk drinking, anxiety + at-risk drinking, or all three problems).

The participants and the RAs performing the baseline and follow-up outcome assessments were blind to the randomization. Participants were informed that they would be randomized and receive one of two interventions of varying intensity, but they were not informed of what the “intensities” were - i.e., eUC consisting of a 45-minute assessment at baseline and after 4, 8, and 12 months vs. tCC consisting of the same assessment plus weekly and then monthly calls (see below). By design, the PCP and the intervention team (i.e., the study psychiatrist and MHT) were informed of the randomization. To preserve the blind for the participant and RAs, all participants were contacted by an MHT, regardless of whether they were randomized to tCC or eUC (see below).

**Active Intervention: Telephone-Based CC Provided by a MHT**

Participants randomized to CC were assigned to a trained bachelor-level research MHT. In most CC studies, this role is held by a clinician (e.g., a psychologist, social worker, or nurse) but several studies have successfully used this type of non-licensed staff or “lay provider”.^12-17^ The MHTs all completed six weeks of training, including relevant readings, observed assessments, and a workshop on the use of the BHL software. In addition, they completed training in motivational interviewing and received weekly case supervision.

After completion of the baseline assessment by the RA, participants randomized to tCC were contacted by the MHT. During this first call, they established rapport, reviewed the results of the baseline assessment, completed an additional assessment addressing past treatment history, recent psychosocial stressors, sleep hygiene, alcohol use, and treatment preference. All this information was reviewed with the study psychiatrist (DJR) during weekly group supervision meetings when a formal treatment plan was designed with pharmacotherapy guided by a treatment algorithm.^1^ Recommendations from this treatment plan were e-faxed to the PCP who was free to implement them, ignore them, or to provide additional “usual care”.

Other than participants with subthreshold symptoms, MHTs provided support to all participants randomized to tCC, including help to identify resources (e.g., websites, apps, community-based services); and regular telephone monitoring and psychoeducation throughout the twelve-month study. Typically, these calls were weekly during the early months and tapered down to monthly during the later months; they lasted 20-40 minutes and started with the administration of the PHQ-9, GAD-7, or an inquiry about the number of daily drinks, as warranted by the problems identified during the baseline assessment. In addition, the MHT monitored treatment adherence and adverse effects. Depending on participants’ readiness, education and support focused on the identified problems, the need for initiating treatment, the rationale for specific treatment recommendations (e.g., an antidepressant recommended for anxiety), or how to prepare for visits with the PCP (e.g., how to request initiation of a recommended antidepressant).

**Control Group: Usual Care Enhanced by Comprehensive Assessments**

Like participants randomized to the intervention, those randomized to the control group (“enhanced usual care” or eUC) were assessed by an RA at baseline and after 4, 8, and 12 months and results of these assessments were shared with PCPs. No recommendations were provided and the PCP was free to manage the participant as they deemed appropriate, including referral to a psychiatrist. As described above, to maintain the blind, participants randomized to EUC were also contacted by an MHT after completion of each assessment by the RA. During these brief calls, the MHT informed participants that the results of their assessment were shared with their PCP and they would be contacted again in four months.

**Outcomes**

The primary outcome measure was the change in PHQ-9 from baseline to 12 months in those presenting with depression. Because of the transdiagnostic recruitment strategy, secondary outcome measures were: change in GAD-7 from baseline to 12 months in those presenting with a anxiety; or change in the number of weekly drinks from baseline to 12 months in those presenting with at-risk drinking. Additional pre-planned outcomes included: changes in health-related quality of life and functioning in the whole sample as measured by the Veterans RAND 12-Item Health Survey (VR-12) at 4, 8, and 12 months; rates of response or remission for depression in the depression group (defined as PHQ-9 score < 10 and < 5, respectively); rates of response or remission for anxiety in the anxiety group (defined as GAD-7 score < 10 and < 5, respectively); and decrease in alcohol use to the point where it meets the guidelines for safe drinking (i.e., no more than 15 drinks per week for men or 10 drinks for women and no more than 4 binges during the past 3 months). As additional explanatory variables, we also assessed the rates of initiation or change in antidepressant medications or psychosocial interventions reported by participants in the whole sample at 4, 8, and 12 months. In addition, we reviewed health records in a subgroup of participants (n=347) and assessed documentation by the PCP of initiation of antidepressants and of adequacy of antidepressant treatment using the Antidepressant Treatment History Form.

**Statistical Analyses**

We compared the changes from baseline to 12 months between the two intervention groups with linear contrasts of estimated means for the three main outcomes measures (PHQ-9 scores, GAD-7 scores, number of daily drinks). Mixed models were used to assess the changes in depression or anxiety scores, and changes in health-related quality of life and functioning. Because the number of drinks is a count variable that is skewed, and mixed model assumes normality, a generalized estimating equation (GEE) with logarithmic link and variance function from the negative binomial distribution was used to assess changes in number of drinks. Both mixed models and GEE were specified with a time by group interaction, where time was considered categorical. Sites and participants within sites were specified as random effects in the mixed models and clusters in the GEE. Because of the high rate of comorbidity, baseline PHQ-9 score, GAD-7 scores and number of drinks per week were adjusted for in the three models. A similar approach was used for the analysis of changes in health-related quality of life and functioning (VR-12 scores) in the whole sample. We also compared categorical outcomes (i.e., rates of remission, response, or safe drinking) by intervention group using logistic regression, after classifying each subject as a responder or remitter at 12 months. Multiple imputation of 50 datasets was conducted in Mplus 8.2^18^ to impute the response or remission status at 12 months for participants who dropped out. Multiple imputation was conducted through variance and covariance modeling^19^, using for the imputation the available demographics and clinical measures at previous time points. The estimation of the study group effect used the option COMPLEX in Mplus, which adjusts the standard error of estimates for the site clustering in the data using the Huber-White sandwich estimator.^20,21^ A logistic regression analysis was used to determine whether the proportion of participants receiving treatment differed in the two groups after controlling for relevant covariates and time to initiation of, or first change in, treatment was compared in the two groups using Cox regression. The Fisher test was used to compare the rates of initiation of antidepressants and the adequacy of antidepressant trials documented in health records. All analyses were conducted using SAS System 9.4, except for the analysis of response and remission outcomes, which were conducted using Mplus.

**Sample Size and Power**

Based on the initial number of participating practices and PCPs, we had planned to randomize 1,000 participants allowing us to detect small effect sizes. Because of the actual low referral rates,^1,2^ we ended up randomizing 502 participants, which should still be enough to detect small effect sizes given the distribution of these participants in each of the three main diagnostic groups. The at-risk drinking group has the smallest number of participants (n = 113) and 113 participants randomized into two groups of equal size provides 80% power to detect a standardized effect size Cohen’s f = 0.13, assuming a Repeated Measure ANOVA targeting a time by group interaction with alpha = 0.05, correlation between measures of 0.3 (which is the lowest intra-class correlation in our data across the three outcomes), and four time points. For the depression group (n = 246), the minimal detectable effect size Cohen’s f = 0.09 and in the anxiety group (n = 194), the minimal detectable effect size Cohen’s f = 0.10.

**Reasons for Low Recruitment Rate**

To better understand the low referral rate to the PARTNERS trial, we conducted a qualitative study of 23 PCPs and their staff involved in the trial.^2^ We used stratified sampling to reach high-, low- and nonreferring providers in urban and rural settings. The qualitative findings revealed that study referral rates were influenced by the limited relationship between PARTNERs MHTs and PCPs, and variable attention to leadership, training and quality improvement as vital elements of collaborative care.

**REFERENCES:**

1. Rodie DJ, Fitzgibbon K, Perivolaris A, Crawford A, Geist R, Levinson A, et al. The primary care assessment and research of a telephone intervention for neuropsychiatric conditions with education and resources study: Design, rationale, and sample of the PARTNERs randomized controlled trial. Contemporary Clinical Trials. 2021 Apr 1;103:106284.
2. Sunderji N, Ion A, Zhu A, Perivolaris A, Rodie D, Mulsant BH (2019). Challenges in conducting research on collaborative mental health care: a qualitative study. CMAJ Open 7(2):E405-E414_doi: 10.9778/cmajo.20180172
3. Zaheer S, Garofalo V, Rodie D, Perivolaris A, Chum J, Crawford A, Geist R, Levinson A, Mitchell B, Oslin D, Sunderji N, Mulsant BH ((2018). Computer-aided telephone support for primary care patients with common mental health conditions: randomized controlled trial. Journal of Medical Internet Research Mental Health 5(4):e10224_doi: 10.2196/10224
4. Kroenke K, Spitzer RL, Williams JBW. The PHQ-9. J Gen Intern Med. 2001 Sep;16(9):606–13.
5. Spitzer RL, Kroenke K, Williams JBW, Löwe B. A brief measure for assessing generalized anxiety disorder: the GAD-7. Arch Intern Med. 2006 May 22;166(10):1092–7.
6. Butt P, Canadian Centre on Substance Abuse. Alcohol and health in Canada: a summary of evidence and guidelines for low-risk drinking [Internet]. Ottawa, Ont.: Canadian Centre on Sustance Abuse; 2012 [cited 2021 Oct 21]. Available from: https://www.deslibris.ca/ID/233659
7. Katzman R, Brown T, Fuld P, Peck A, Schechter R, Schimmel H. Validation of a short Orientation-Memory-Concentration Test of cognitive impairment. Am J Psychiatry. 1983 Jun;140(6):734–9.
8. Paykel ES, Myers JK, Lindenthal JJ, Tanner J. Suicidal feelings in the general population: a prevalence study. Br J Psychiatry. 1974 May;124(0):460–9.
9. Weathers FW, Litz BT, Keane TM, Palmieri PA, Marx BP, Schnurr PP. The PTSD Checklist for DSM-5 (PCL-5). 2013. Scale available from the National Center for PTSD at [www.ptsd.va.gov](http://www.ptsd.va.gov).
10. Harris PA, Taylor R, Thielke R, Payne J, Gonzalez N, Conde JG. Research electronic data capture (REDCap)--a metadata-driven methodology and workflow process for providing translational research informatics support. J Biomed Inform. 2009 Apr;42(2):377–81.
11. Kazis LE, Miller DR, Skinner KM, Lee A, Ren XS, Clark JA, et al. Applications of methodologies of the Veterans Health Study in the VA healthcare system: conclusions and summary. J Ambul Care Manage. 2006 Jun;29(2):182–8.
12. Chibanda D, Verhey R, Munetsi E, Rusakaniko S, Cowan F, Lund C. Scaling up interventions for depression in sub-Saharan Africa: lessons from Zimbabwe. Glob Ment Health (Camb). 2016;3:e13.
13. Patel V, Weiss HA, Chowdhary N, Naik S, Pednekar S, Chatterjee S, et al. Effectiveness of an intervention led by lay health counsellors for depressive and anxiety disorders in primary care in Goa, India (MANAS): a cluster randomised controlled trial. Lancet. 2010 Dec 18;376(9758):2086–95.
14. Stimpson A, Kroese BS, MacMahon P, Rose N, Townson J, Felce D, et al. The experiences of staff taking on the role of lay therapist in a group-based cognitive behavioural therapy anger management intervention for people with intellectual disabilities. J Appl Res Intellect Disabil. 2013 Jan;26(1):63–70.
15. Shahmalak U, Blakemore A, Waheed MW, Waheed W. The experiences of lay health workers trained in task-shifting psychological interventions: a qualitative systematic review. International Journal of Mental Health Systems. 2019 Oct 14;13(1):64.
16. Stanley MA, Wilson NL, Amspoker AB, Kraus-Schuman C, Wagener PD, Calleo JS, et al. Lay providers can deliver effective cognitive behavior therapy for older adults with generalized anxiety disorder: a randomized trial. Depress Anxiety. 2014 May;31(5):391–401.
17. Gensichen J, von Korff M, Peitz M, Muth C, Beyer M, Güthlin C, et al. Case management for depression by health care assistants in small primary care practices: a cluster randomized trial. Ann Intern Med. 2009 Sep 15;151(6):369–78.
18. Muthén LK, Muthén BO. Mplus User’s Guide. Seventh Edition. Los Angeles: CA: Muthén & Muthén; 1998.
19. Asparouhov T, Muthen B. Multiple Imputation with Mplus. :25.
20. Huber PJ. The behavior of maximum likelihood estimates under nonstandard conditions. Proceedings of the Fifth Berkeley Symposium on Mathematical Statistics and Probability, Volume 1: Statistics. 1967 Jan 1;5.1:221–34.
21. White H. A Heteroskedasticity-Consistent Covariance Matrix Estimator and a Direct Test for Heteroskedasticity. Econometrica. 1980;48(4):817–38.
